# Clinical and genetic spectrum of primary ciliary dyskinesia in Chinese patients: a systematic review

**DOI:** 10.1186/s13023-022-02427-1

**Published:** 2022-07-19

**Authors:** Bo Peng, Yong-hua Gao, Jia-qi Xie, Xiao-wen He, Cong-cong Wang, Jin-fu Xu, Guo-jun Zhang

**Affiliations:** 1 Department of Respiratory and Critical Care Medicine, The First Affiliated Hospital of Zhengzhou University, 1 Jianshe East Road, Zhengzhou, 450052 Henan China; 2 Department of Respiratory and Critical Care Medicine, Shanghai Pulmonary Hospital, Tongji University School of Medicine, No. 507 Zhengmin Road, Shanghai, 200433 China; 3 Department of Respiratory and Critical Care Medicine, Xiangyang Central Hospital, Xiangyang, China; 4 Department of Respiratory and Critical Care Medicine, Xuchang Central Hospital, Xuchang, China

**Keywords:** PCD, Systematic review, Genotype, Phenotype, Cilia

## Abstract

**Background:**

Primary ciliary dyskinesia (PCD) represents a highly heterogenous disorder with extensive clinical and genetic patterns among populations of different geographic location and ethnic origin. However, data about Chinese patients are limited. We aimed to summarize the clinical and genetic spectrum of Chinese PCD patients based on all available literatures.

**Methods:**

We searched Embase, Pubmed, Web of Science and Chinese databases including CNKI, SinoMed and Wanfang from 1981 to 2021, to identify articles reporting patients with PCD in China, which had included information about transmission electron microscopy and/or genetic testing.

**Results:**

A total of 244 Chinese PCD patients in 52 articles were included. Of these patients, the mean age was 13.1 years, and 55 patients (22.5%) were diagnosed with PCD after 18 years old. Compared with patients diagnosed with PCD in childhood or infancy, patients diagnosed with PCD in adulthood had a higher prevalence of chronic wet cough, sinusitis, *Pseudomonas aeruginosa* (PA) isolation and radiological bronchiectasis as well as worse lung function. 25 PCD-related genes were identified in 142 patients, and DNAH5, DNAH11, CCDC39 and CCDC40 were the most frequently detected mutations. More than half of genetic variants were loss-of-function mutations, and the majority of these variants were seen only once. Correlations between PCD phenotype, genotype and ciliary ultrastructure were also evidenced.

**Conclusions:**

Diagnostic delay and under-recognition of PCD remain a big issue in China, which contributes to progressive lung disease and PA infection indicating worse outcome. Specialist equipment and expertise are urgently required to facilitate the early diagnosis and treatment of PCD.

***Trial registry*:**

PROSPERO; No.: CRD42021257804; URL:www.crd.york.ac.uk/prospero/

**Supplementary Information:**

The online version contains supplementary material available at 10.1186/s13023-022-02427-1.

## Introduction

Primary ciliary dyskinesia (PCD, OMIM ID: 244,400) is a rare autosomal-recessive or X-linked disorder caused by mutations in genes that encode the specific structure or function of motile cilia [1]. Characteristics of PCD are clinically and genetically heterogeneous, but all associated with abnormal motile ciliary function. Patients with PCD typically manifest with unexplained neonatal respiratory distress syndrome (NRDS), chronic wet cough, sinusitis, otitis media, laterality disorders, infertility and almost 100% concomitant with bronchiectasis in adulthood [2, 3].

As with many rare diseases, the diagnosis of PCD is challenging and requires a multi-test diagnostic approach since no single test has adequate sensitivity or specificity [4]. Both the European Respiratory Society (ERS) and the American Thoracic Society (ATS) guidelines agreed that a diagnosis of PCD can be ascertained if a hallmark ciliary ultrastructural defect observed by transmission electron microscopy (TEM) or biallelic pathogenic variants in a known PCD gene were identified, with nasal nitric oxide (nNO) being recommended as the initial diagnostic tool in light of its costs and accessibility [5, 6]. Recently, delineation of genotype–phenotype relationships has been emerged in patients with PCD [7]. For example, patients with absent inner dynein arm with microtubular disorganization who harbor a CCDC39 or CCDC40 mutation, have worse lung function compared with patients with outer dynein arm defects and DNAH5 mutation. In contrast, patients with RSPH1 mutations appear to have mild lung function impairment [8, 9]. However, caution is needed when interpreting these genotype–phenotype data based on a small number of patients.

The estimated prevalence of PCD is 1:10,000–1:20,000 in Europeans, while the higher prevalence has been reported in the British Asian population [7, 10]. Considering the large population of China and high prevalence of bronchiectasis, PCD patients might be not as rare as we previously thought [11]. However, studies about PCD in China are mainly from case report and small case series due to multiple factors, including but not limited to a low awareness of PCD amongst physicians, highly heterogeneous symptoms of PCD, the lack of diagnostic facilities as well as the existing misconception that identification of PCD makes a little difference to the management of patients with bronchiectasis. Recently, several relatively larger case series have showed that the clinical manifestations, ciliary phenotypes, and genetic spectrum of Chinese PCD are highly diverse and might have some distinct features compared to those observed in Caucasians, which has enhanced our understanding about PCD in Chinese patients [12, 13]. Interestingly, one recent study in PCD has revealed a striking genetic stratification through multigene panel by next-generation sequencing according to the population of different origins [14]. This highlights the importance of describing the clinical characteristics and genetic spectrums of PCD in different geographic and ethnic regions, including in China, which could have important implications for clinical management and future research.

To raise awareness and improve management of PCD in China, we conducted a systematic review aiming to: (1) summarize the clinical manifestations, ciliary phenotype and mutation spectrum of Chinese PCD patients; (2) delineate the relationships between genotype, phenotype and ciliary ultrastructure, based on all available data.

## Methods

### Data sources and search strategy

Embase, Pubmed, Web of Science and Chinese databases including CKNI, SinoMed and Wanfang were searched electronically to identify studies reporting PCD cases in Chinese patients published in English or Chinese from January 1981 to May 2021. The search strategy included the following key terms: (‘primary ciliary dyskinesia’ OR ‘Kartagener syndrome’ OR ‘immotile cilia syndrome’) AND (‘Chinese’ OR ‘China’ OR ‘Han population’). The electronic searches were supplemented by manual screening of the reference lists of all accepted articles to identify additional studies which were not included in the initial search. We restricted our search to articles published since 1981, as older studies are rarely available online and many things have changed for the diagnostic modalities of PCD, with TEM and/or genetic test being increasingly used. [15]

### Definition of PCD patients

Based on current guidelines, the diagnosis of PCD can be confirmed by the identification of a hallmark defect of ciliary ultrastructure observed by TEM or bi-allelic pathogenic mutations in a known PCD gene, with measurement of nNO being recommended as a screening tool. Kartagener’s syndrome is a type of PCD that is characterized by situs inversus, chronic sinusitis, and bronchiectasis [5, 6]. To better understand the relationship between phenotype, ciliary ultrastructure and genotype in Chinese patients, we only included studies which have reported the TEM and/or genetic results. The diagnostic strategies for PCD were determined by the authors in each study.

### Article selection

Two investigators (BP and JQX) independently screened the eligible studies based on title and abstract of all identified articles according to the predefined inclusion and exclusion criteria, with discrepancies resolved by consensus discussion of BP, JQX and YHG. Studies were included if they met all the following criteria: (1) diagnosis of PCD, Kartagener syndrome or immotile cilia syndrome; (2) patients with Chinese origin; 3) available TEM and/or genetic data. The exclusion criteria included: (1) wrong study type (i.e. reviews, conference abstracts, commentaries and editorials); (2) studies without peer review. Patients overlap among studies are likely to exist because most studies on PCD are performed in a limited number of centers in China. When PCD patients are reported more than once by the same center with overlapping inclusion periods, only the largest studies were included.

### Data extraction

Data was extracted from all eligible articles using standardized excel forms. Data retrieved from the studies were as follows: (1) study characteristics: paper’s title, first author, year of publication, study location, the number of PCD patients; (2) baseline information of patients: gender, age at onset of symptoms, age at diagnosis, family history; (3) clinical characteristics: history of NRDS, respiratory symptoms, sinus disease, otitis media, hearing impairment, viscera situs, infertility and other comorbidities; (4) laboratory investigations: spirometry, respiratory culture, nNO measurement, TEM analysis, high-speed video analysis (HSVA), saccharin test, immunofluorescence test, genetic testing and radiological features. The methods for medical history ascertainment were determined by the authors in each study. Regarding past history, symptoms, and comorbidities, we opted to record as “no” if it was not reported in the included studies. The age at onset of symptoms was not clearly described in many articles, instead of using terms such as “about 10 years ago” or “childhood”, as an alternative. We therefore categorized patients into three groups about the age at onset of symptoms: infancy (< 1 year), childhood (1–17 years) and adulthood (≥ 18 years).

### Statistical analysis

Data analysis was performed using SPSS (version 22), and GraphPad Prism (version 6.07). Data were presented as mean (standard deviation, SD), median (interquartile range, IQR) or count (proportion), as appropriate. Categorical variables were compared using chi-squared test. Continuous variables were compared using unpaired t tests or Mann–Whitney test when appropriate. All analyses were two-sided with *P* < 0.05 considering as a statistical significance. The review protocol was prospectively registered at PROSPERO, number CRD42021257804.

## Results

### Search attrition

**Fig. 1:**
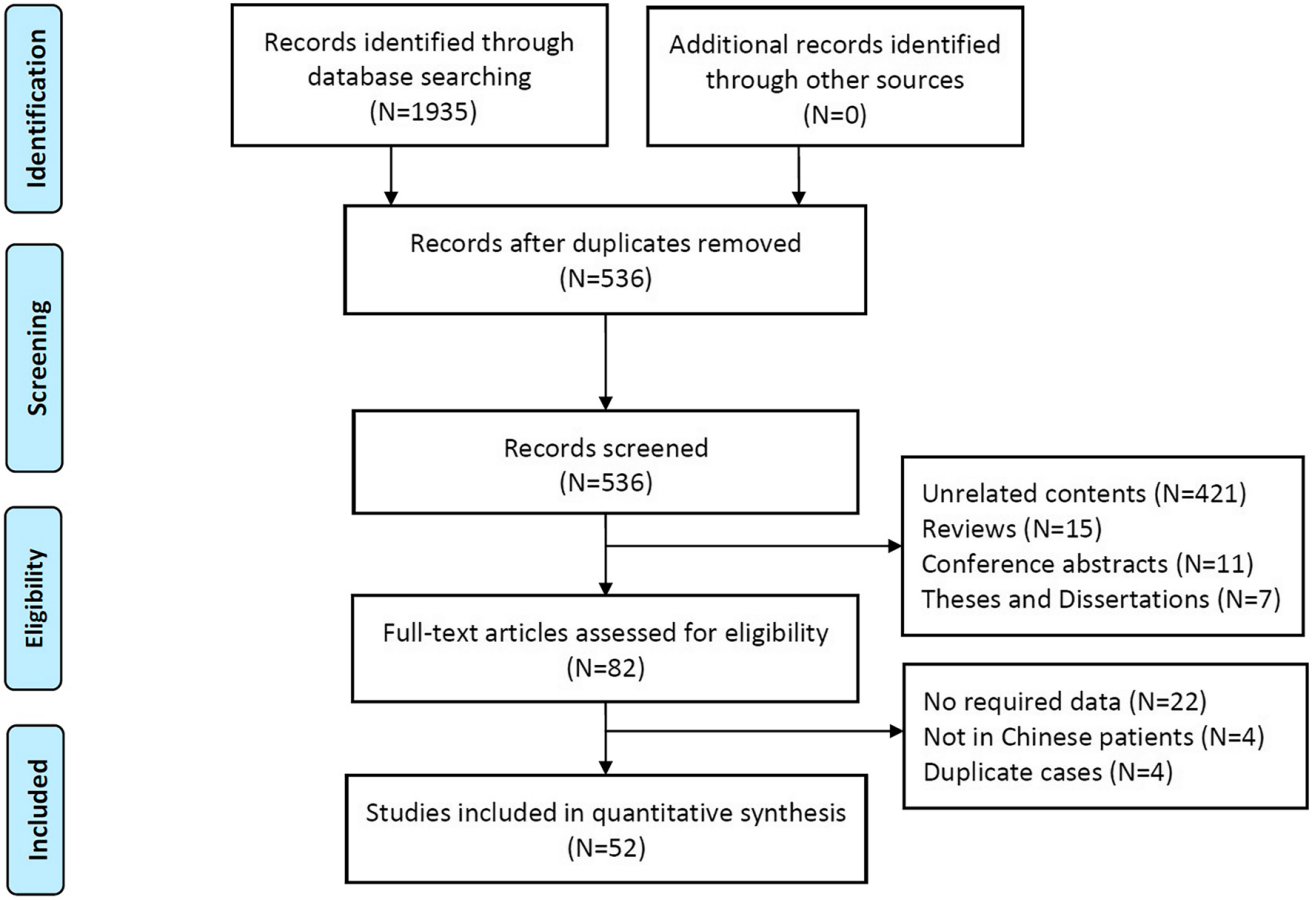
A flow chart showing the procedure for identifying the studies included in the systematic review

**Fig. 2:**
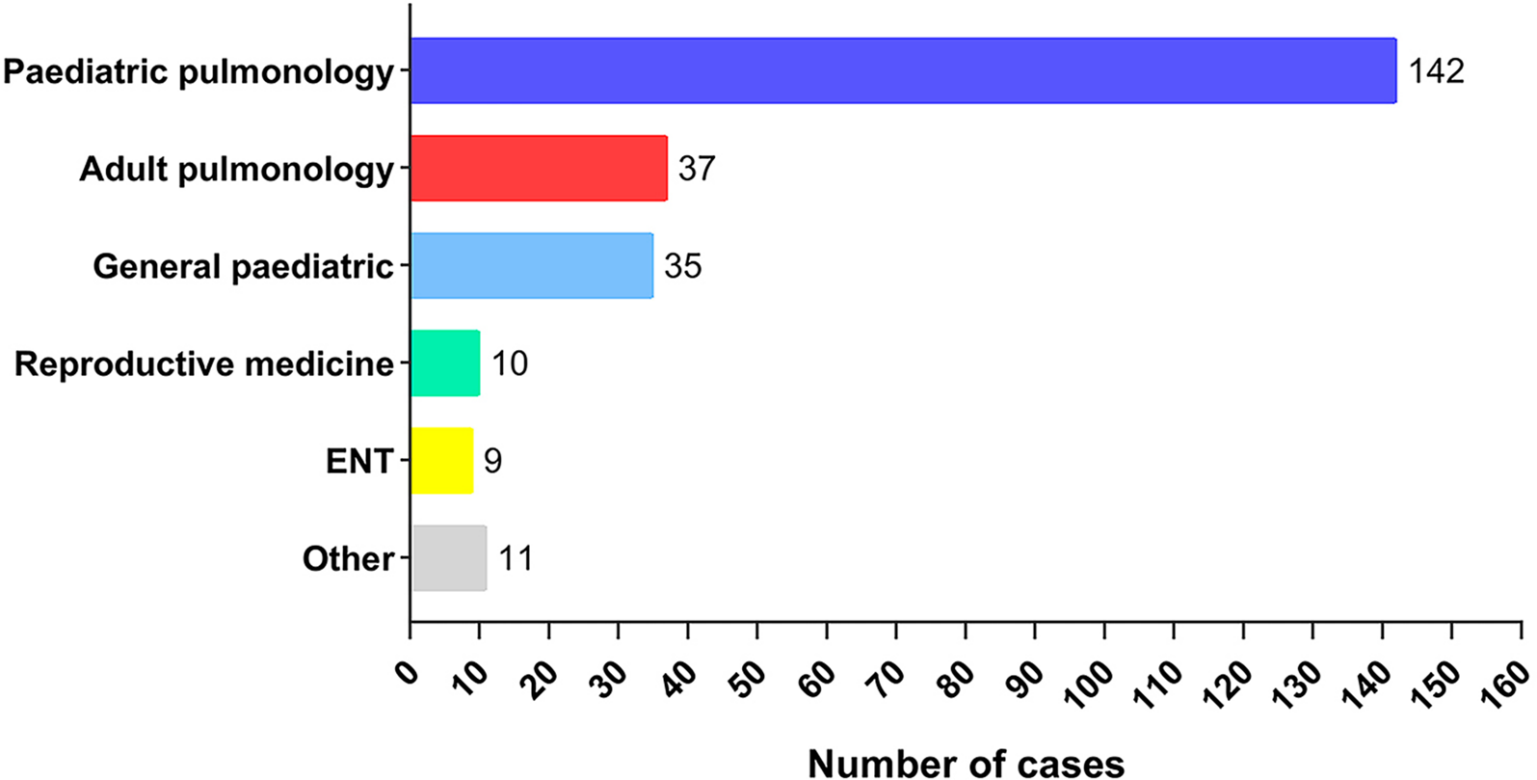
Department distribution of diagnosed PCD and the number of reported cases. ENT = ear-nose-throat

The process of inclusion and exclusion of studies was shown in Fig. 1. We extracted 1935 abstracts or articles after retrieval, and 536 were screened for eligibility while 1399 duplicate documents were removed. Upon further review, 454 records were excluded according to the title and abstract. Of 82 articles for full-text review, 52 articles (24 in English and 28 in Chinese) with 244 PCD patients were included in the final analysis, [12, 13, 16–65] and the excluded articles are listed in online supplement (Additional file 1: Table S1). Among them, only 3 articles reported 10 or more patients [12, 13, 43], and the remaining were case reports or small case series [16–42, 44–65]. Geographically, 5 patients were from Hong Kong, one from Taiwan, and the remaining from mainland China. Interestingly, pediatric department reported 72.5% of the PCD cases whilst adult pulmonology reported only 15.2% of the cases (Fig. 2). The study characteristics of each included articles are listed in online supplement (Table S2 is changed to Additional file 1: Table S2). The clinical characteristics and laboratory investigations of patients who had a clinical diagnosis of PCD in excluded articles after full text review were summarized in Table S2 is changed to Additional file 1: Table S3 (online supplement for details).

### Clinical manifestations of Chinese patients

Demographic and clinical characteristics of 244 PCD patients were summarized in Table 1. Of these patients, 116 (47.5%) were female, 120 (49.2%) had situs inversus totalis or heterotaxy and 189 (77.5%) were diagnosed with PCD before 18 years old. With regard to the age of symptom onset, 120 (49.2%) were in their infancy, 85 (34.8%) in childhood and 3 (1.2%) in adulthood. In 237 patients whose age at diagnosis was clearly stated, the mean age was 13.1 years old. Of 55 adult patients, 23 (41.8%) had a history of infertility. The common manifestations were chronic wet cough (92.2%), rhinosinusitis (84.0%), otitis media (27.5%), hearing impairment (20.9%) and the common comorbidities were asthma (11.5%), thoracic deformity (7.0%), congenital heart disease (4.5%), and gastroesophageal reflux (3.7%). Other comorbidities included post infectious bronchiolitis obliterans (2.5%), congenital deafness (1.2%) and diffuse pan-bronchiolitis (0.8%).

**Table 1:** Clinical characteristics and laboratory results of Chinese PCD patients categorized by age at diagnosis

Parameters	Total (n = 244)	Age at diagnosis < 18 n = 189	Age at diagnosis ≥ 18 n = 55	*P* values
Age at diagnosis (n = 237), mean (SD)	13.1 ± 11.3	8.2 ± 4.1(n = 189)	32.1 ± 10.1(n = 48)	< 0.0001
Gender, female (%)	116(47.5)	90 (47.6)	26 (47.3)	0.96
Age at symptom onset				
Infancy	120 (49.2)	112 (59.3)	8 (14.5)	< 0.0001
Childhood	85 (34.8)	47 (24.9)	38 (69.1)	< 0.0001
Adulthood	3(1.2)	NA	3 (5.5)	NA
NA	36 (14.8)	30 (15.9)	6 (10.9)	0.36
Situs inversus totalis/heterotaxy	120 (49.2)	82 (43.4)	38 (69.1)	0.001
Family history				
Consanguineous patients	24 (9.8)	4 (2.1)	20 (36.4)	< 0.0001
PCD family history	26 (10.7)	11 (5.8)	15 (27.3)	< 0.0001
Infertility history				
Female	7 (2.9)	NA	7 (12.7)	NA
Male	16 (6.6)	NA	16 (29.1)	NA
Symptoms and Comorbidities				
Neonatal respiratory distress	60 (24.6)	60 (31.8)	0 (0)	< 0.0001
Chronic wet cough	225 (92.2)	170 (89.9)	55 (100)	0.03
Sinusitis	205 (84.0)	153 (81.0)	52 (94.5)	0.02
Otitis media	67 (27.5)	59 (31.2)	8 (14.6)	0.02
Hearing impairment	51 (20.9)	40 (21.2)	11 (20.0)	0.85
Asthma	28 (11.5)	22 (11.6)	6 (10.9)	0.88
Congenital heart disease	11 (4.5)	10 (5.3)	1 (1.8)	0.47
Thoracic deformity	17 (7.0)	16 (8.5)	1 (1.8)	0.16
Diffuse panbronchiolitis	2 (0.8)	0 (0)	2 (3.6)	0.05
Post infectious bronchiolitis obliterans	6 (2.5)	6 (3.2)	0 (0)	0.34
Gastroesophageal reflux	9 (3.7)	9 (4.8)	0 (0)	0.21
Congenital deafness	3 (1.2)	2 (1.1)	1 (1.8)	0.54
Nasal NO test (n = 102)				
Below diagnostic cut-off	93 (91.2)	89 (93.3)	9 (75)	0.12
Respiratory culture (n = 133)		119	14	
* Haemophilus influenzae*	22 (16.5)	22 (18.6)	0 (0)	0.17
* Pseudomonas aeruginosa*	26 (19.5)	18 (15.1)	8 (57.1)	0.001
* Streptococcus pneumoniae*	36 (27.1)	36 (30.3)	0 (0)	0.04
* Streptococcus viridans*	1 (0.8)	0 (0)	1 (7.1)	0.11
* Staphylococcus aureus*	7 (5.3)	6 (5.0)	1 (7.7)	0.55
* Moraxella catarrhalis*	4 (3.0)	4 (3.4)	0 (0)	1
* Legionella pneumophila*	1 (0.8)	1 (0.8)	0 (0)	1
* Acinetobacter baumannii*	1 (0.8)	1 (0.8)	0 (0)	1
* Monilia albican*	2 (1.5)	2 (1.7)	0 (0)	1
* Aspergillus*	1 (0.8)	0 (0)	1 (7.1)	0.11
normal flora	35 (26.3)	32 (26.9)	3 (21.4)	0.91
Radiographic feature (n = 233)		178	55	
Bronchiectasis	164 (70.4)	110 (61.8)	54 (98.2)	< 0.0001
Atelectasis	88 (37.8)	85 (47.8)	3 (5.5)	< 0.0001
Diffuse nodules	29 (12.5)	22 (12.4)	7 (12.7)	0.94
Pneumonia	19 (8.2)	15 (8.4)	4 (7.3)	1
Nearly normal	7 (3.0)	7 (4.0)	0 (0)	0.29
Lung function				
FEV_1_% of predicted, mean (SD)	70.6 (25.5) (n = 45)	83.7 (15.6) (n = 27)	51.0 (25.1) (n = 18)	< 0.0001
FVC % of predicted, mean (SD)	80.9 (20.6) (n = 32)	82.3 (17.5) (n = 25)	75.9 (30.6) (n = 7)	0.61

Abbreviations: *FEV*_*1*_ Forced expiratory volume in one second, *FVC* Forced vital capacity, *PCD* Primary ciliary dyskinesia, *NA* Not available, *NO* Nitric oxide, *SD* Standard deviation

Microbiological results were available in 133 patients. The most commonly isolated pathogens were *Streptococcus pneumoniae* (27.1%), followed by *Pseudomonas aeruginosa* (19.5%), *Haemophilus influenza* (16.5%), and *Staphylococcus aureus* (5.3%). Of 233 patients who had radiographic data, 164 had bronchiectasis, 88 had atelectasis and 29 had diffuse nodules. Regarding lung function, the mean forced expiratory volume in one second (FEV_1_) was 70.6% of the predicted value in 45 patients.

Compared with patients who were diagnosed with PCD before 18 years old, patients diagnosed with PCD in adulthood had a higher prevalence of PCD family history, chronic wet cough, sinusitis, diffuse pan-bronchiolitis, *Pseudomonas aeruginosa* infection, radiological bronchiectasis and had a worse lung function, with contrasting lower prevalence of NRDS, otitis media, *Streptococcus pneumoniae isolate* and radiological atelectasis.

### Genetic spectrums and ciliary characteristics

**Fig. 3:**
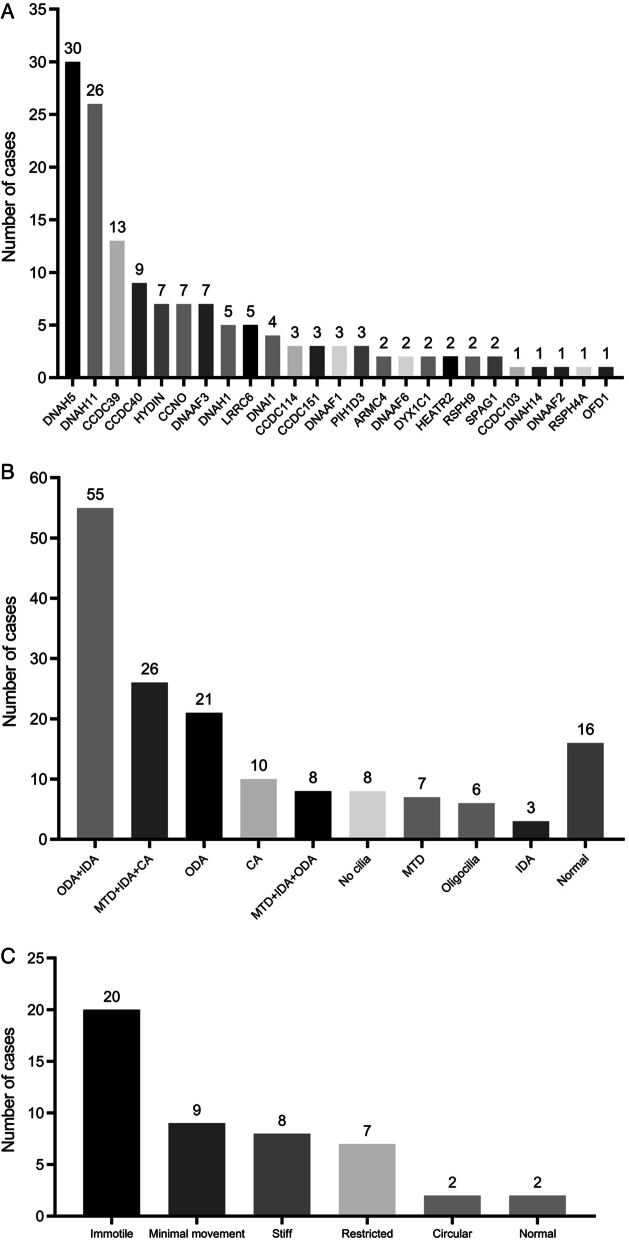
A-Distribution of genetic mutations in 142 cases; B-Distribution of TEM analysis in 160 cases; C-Distribution of HSVA analysis in 48 cases. TEM = transmission electron microscopy; HSVA = high-speed video analysis; ODA = outer dynein arms; IDA = inner dynein arms; MTD = microtubular disarrangement; CA = central apparatus

Regarding genetic spectrum, 25 PCD-related genes were identified in 142 individuals. Of these patients, 105 had compound heterozygous mutations, 27 had homozygous mutations, 6 had X-linked recessive gene mutations and 4 had no gene mutations. Among all the variants, more than half of these variants were loss of function mutations, including frameshift (26.7%), nonsense (21.0%), splicing (9.9%) and deletion (2.9%). DNAH5, DNAH11, CCDC39, CCDC40, HYDIN, CCNO and DNAAF3 were the most frequently identified genes, with a prevalence of 21.1%, 18.3%, 9.2%, 6.3%, 4.9%, 4.9% and 4.9%, respectively, as showed in Fig. 3A.

Of 160 patients who had TEM data, the absence of the outer and inner dynein arm (ODA/IDA), absence of outer dynein arm (ODA), and the microtubular disorganization with defects of inner dynein arm and central apparatus (MTD/IDA/CA) were detected in 55 (34.4%), 21 (13.1%), and 26 (16.3%) cases, respectively (Fig. 3B). Other abnormal ciliary ultrastructure include CA defects (n = 10), MTD with outer and inner dynein arm defects (n = 8), no cilia (n = 8), MTD (n = 7), oligocilia (n = 6) and IDA defects (n = 3). 16 patients (10.0%) present nearly normal cilia.

As for ciliary beat patterns detected by HSVA (n = 48), immotile, minimal movement, stiff, restricted, circular and normal cilia were reported in 20 (41.7%), 9 (18.8%), 8 (16.7%), 7 (14.6%), 2 (4.2%) and 2 (4.2%) cases, respectively. (Fig. 3C).

### Associations between genotype, clinical characteristics and ciliary ultrastructure

The clinical and ciliary ultrastructural data of patients with PCD caused by different genes were showed in Table 2. To summarize, patients with DNAH5 mutation mainly presented ODA defects (75%) by TEM and immotile cilia by HSVA, and patients with DNAH11 mutation had nearly normal ultrastructure (64.3%) by TEM and restricted cilia by HSVA Meanwhile, patients with CCDC39 and CCNO mutation had worse lung function compared with patients who had DNAH5 mutation. By TEM analysis, patients with CCDC39 and CCDC40 mutation had MTD/IDA/CA defects.

**Table 2:** Clinical data, ciliary ultrastructural defects, and genetic mutations in Chinese patients with PCD

GENE	Zygosity	Type of variants	FEV_1_% of predicted	nNO	TEM	Viscera situs	Infertility	Predominant HSVA
DNAH5 (n = 30)	Homo (n = 3)	Missense 3	94.7 (10.9) n = 17	12.6 (5.1) n = 17	ODA (12) ODA + IDA (3) Normal (1)	SIT (16) SS (4) HTX (1)	1	Immotile (11)
	Comp het (n = 25)	Missense 17 nonsense 13 frameshift 12 splicing 5 duplication/deletion/non-coding 1						
DNAH11 (n = 26)	Comp het (n = 25)	Missense 32 nonsense 9 frameshift 4 splicing 3 deletion 1	89.3 (29.3) n = 10	13.1 (8.2) n = 15	Normal (9) Oligocilia (4) CA (1)	SIT (6) SS (3) HTX (2)		Restricted (8)
CCDC39 (n = 13)	Homo (n = 3)	Frameshift 3	62.5 (20.2) n = 10	17.4 (8.4) n = 9	MTD + IDA + CA (10)	SIT (6) SS (6) HTX (1)		Stiff (3) Immotile (2)
	Comp het (n = 10)	Frameshift 9 nonsense 6 splicing 5						
CCDC40 (n = 9)	Comp het (n = 8)	Nonsense 6 missense 4 frameshift 3 deletion 2 splicing 1	94.7 (10.1) n = 3	13.9 (6.7) n = 4	MTD + IDA + CA (7) IDA + MTD + ODA (1)	SIT (7) SS (1)	4	Stiff (4)
HYDIN (n = 7)	Comp het (n = 7)	Missense 6 nonsense 4 splicing 3 frameshift 1			CA (3) MTD (2) Normal (1)	SS (6) HTX (1)		Circular (2)
CCNO (n = 7)	Homo (n = 4)	Frameshift/nonsense 2	62.3 (17.8) n = 6	27.1 (16.7) n = 5	No cilia (2)	SS (4)		
	Comp het (n = 3)	Frameshift 4 nonsense/missense 1						
DNAAF3 (n = 7)	Homo (n = 1)	Missense 1	91.8 (4.9) n = 2	8.2 (7.0) n = 4	ODA + IDA (6)	SIT (5)		Immotile (5)
	Comp het (n = 6)	Frameshift 6 missense 4 splicing/nonsense 1						
DNAH1 (n = 5)	Homo (n = 2)	Nonsense 2		37.1 (16.1) n = 4	Normal (1)	SIT (1)	1	
	Comp het (n = 3)	Missense 4 frameshift/splicing 1						
LRRC6 (n = 5)	Homo (n = 3)	Frameshift/nonsense/deletion 1		8.7 n = 1	ODA + IDA (2)	SIT (3) SS (1)	2	
	Comp het (n = 2)	Missense 3 splicing 1						
DNAI1 (n = 4)	Homo (n = 2)	Missense 2	61.4 (24.2) n = 2	5.8 (1.0) n = 2	ODA (3)	SIT (2)	1	Immotile (1)
	Comp het (n = 2)	Missense 3 Frameshift 1						
CCDC114 (n = 3)	Homo (n = 1)	Missense 1	100.9 (n = 1)	12.1 (n = 1)		SS (1)		Stiff (1)
	Comp het (n = 2)	Frameshift/splicing 2						
CCDC151 (n = 3)	Homo (n = 2)	Frameshift/nonsense 1				SIT (3)		
	Comp het (n = 1)	Frameshift/splicing 1						
DNAAF1 (n = 3)	Homo (n = 1) Comp het (n = 2)	Nonsense 1 Frameshift 3 splicing 1	38.4(20.7) n = 2	14 (n = 1)	ODA + IDA (1)	SIT (3)	2	Immotile (1)
PIH1D3 (n = 3)	X-linked (n = 3)	Deletion 2 frameshift 1			ODA + IDA (2)	SIT (1) SS (1) HTX (1)	2	
ARMC4 (n = 2)	Homo (n = 1)	Frameshift 1	32 (n = 1)	7.1 (n = 1)	ODA (1)	SIT (1)		
	Comp het (n = 1)	Missense 2						
DNAAF6 (n = 2)	X-linked (n = 2)	Frameshift/missense 1			ODA + IDA (2)	SS (1) HTX (1)	2	
DYX1C1 (n = 2)	Homo (n = 2)	Nonsense 2	44 (n = 1)			SIT (2)	2	
HEATR2 (n = 2)	Comp het (n = 2)	Missense 3 synonymous 1			ODA + IDA (1)			
RSPH9 (n = 2)	Comp het (n = 2)	Missense 3 non-coding 1			CA (1)			
SPAG1 (n = 2)	Comp het (n = 2)	Frameshift 4			ODA + IDA (2)	SIT (1)		
CCDC103 (n = 1)	Homo (n = 1)	Frameshift 1		9.5 (n = 1)	ODA (1)	SIT (1)		Immotile (1)
DNAH14 (n = 1)	Comp het (n = 1)	Frameshift 2		63 (n = 1)	IDA (1)			
DNAAF2 (n = 1)	Comp het (n = 1)	Nonsense 2				SIT (1)	1	
OFD1 (n = 1)	X-linked (n = 1)	frameshift 1		6.2 (n = 1)	IDA + MTD + ODA (1)	SIT (1)		Stiff (1)
RSPH4A (n = 1)	Homo (n = 1)	Missense 1			CA (1)			

Abbreviations: *FEV*_*1*_ Forced expiratory volume in one second, *nNO* Nasal nitric oxide, *TEM* Transmission electron microscopy, *HSVA* High-speed video analysis, *ODA* Outer dynein arms, *IDA* Inner dynein arms, *MTD* Microtubular disarrangement, *CA* Central apparatus, *SIT* Situs inversus totalis, *HTX* Heterotaxy, *SS* Situs solitus, *Homo* Homozygous, *Comp* *het* Compound heterozygous

## Discussion

Our study, for the first time, systematically summarized the clinical manifestations, genotype and ciliary ultrastructure of Chinese PCD patients based on current literatures. We show that there is a significantly delayed diagnosis of PCD possibly due to low awareness and limited diagnostic techniques, contributing to progressive lung disease and *Pseudomonas aeruginosa* infection. Overall, mutation spectrums of Chinese PCD patients were similar with those in western countries except higher detection rate of HYDIN mutation. The correlations between genotype, clinical phenotype and ciliary ultrastructure were also evidenced based on small sample size.

**Fig. 4:**
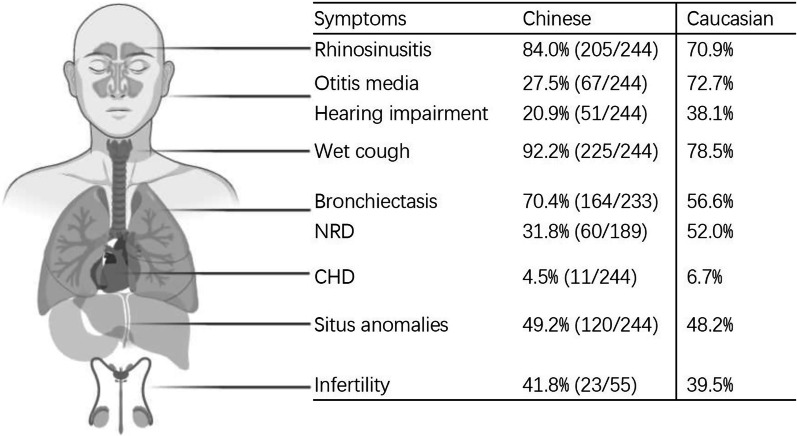
Comparison of clinical manifestations between Chinese and Caucasian patients with PCD. NRD = neonatal respiratory distress; CHD = congenital heart disease

Clinical features of Chinese PCD patients are similar to those in Caucasian patients including chronic wet cough, sinusitis, chronic middle ear disease with or without hearing loss, situs anomalies and history of NRDS  (Fig. 4) [66]. It seems that lower prevalence of middle ear disease was reported in Chinese PCD patients compared with that in western countries (27.5% vs. 72.7%). The possible explanations include the lack of multidisciplinary team for management of PCD in China and insidious middle ear disease being overshadowed by their upper or lower airway symptoms.

The mean age of patients at diagnosis was 13.1 years old, suggesting a significantly delayed diagnosis of PCD in China [67]. The reasons for a diagnostic delay were speculated to be multifactorial, but at least including the following aspects: (1) the misconception that a diagnosis of PCD makes little difference to the management of patients with bronchiectasis; (2) the low awareness of PCD among physicians, especially for patients without situs anomalies; (3) the lack of access to specific diagnostic facilities and expertise in vast majority of healthcare settings; (4) the expensive costs of current diagnostic tests. In addition, although the identification of PCD was advocated in adult bronchiectasis guidelines when patients have clinical features compatible with this disease [68], only 37 cases were reported at adult pulmonary department to date, indicating huge under-recognition of PCD in adults in view of higher prevalence of bronchiectasis and huge population in China. These under-recognition and delayed diagnosis of PCD are inevitably associated with adverse health outcomes, such as an impairment of lung function, and increased likelihood of *Pseudomonas aeruginosa* infection and presence of bronchiectasis on chest image, which is evidenced in our study.

Extensive genetic heterogeneity in PCD was noticed in recent years with over 40 causative genes being identified. Mutations in 5 genes (DNAH5, DNAH11, DNAI1, CCDC39 and CCDC40) are the most common type of mutations in western countries although the vast majority of variants are private mutations [2]. Our findings are consistent with previous studies except that HYDIN other than DNAI1 is more common in Chinese patients. Mutations in HYDIN are known to cause CA defects, in which patients had very subtle ciliary beating abnormalities, nearly normal ciliary ultrastructure and situs solitus, making patients with CA defects difficult to diagnose. Owing to a recent evolutionary event, HYDIN became duplicated and therefore most of the coding exons of HYDIN are also present in the pseudogene HYDIN2 [69, 70]. Because of this duplicated DNA sequence, genetic analysis of HYDIN mutations is complicated, some commercial genetic kits available in the US or Europe do not screen for HYDIN mutations or have limited test sensitivity, highlighting potential cases at risk of being missed by standard functional tests in PCD patients carrying this genetic mutation [7]. These inherent factors, in combination with potential selected bias based on the small number of cases in our systematic review, may help to explain the differences. Noticeably, DNAH14 as a PCD-related gene was first reported in Chinese patients, which broadened the genetic spectrums of PCD [36].

To date, associations between genotype, phenotype and ciliary ultrastructure have been emerged. Gene mutations that encode ciliary proteins would result in functional defects in ODA, ODA docking, preassembly factors, axonemal ruler, nexin link dynein regulator complex proteins (N-DRCs), RS components and CA associated proteins [7, 71]. Our results are consistent with this principle. In addition, lung function in patients with PCD are significantly heterogenous, ranging from normal to very severe impairment [72, 73]. Previous studies have showed that patients with CCNO mutations would experience a rapid deterioration of lung function [74], and patients with CCDC39 or CCDC40 mutations had worse lung function and MTD/IDA/CA defects by TEM analysis [8]. Our results supported these findings except of nearly normal lung function in patients with CCDC40 mutations. However, only 3 children had a CCDC40 mutation, which might bias the result due to limited cases. Further large-scale cohort studies are essential to better elucidate the genotype–phenotype relationships.

Only 41.8% participants have been undergone the nasal NO test in China. As a noninvasive, affordable and relatively easy test, nasal NO should be strongly advocated in the etiological evaluation of bronchiectasis when indicated. In addition, the accessibility and costs of genetic tests have been largely improved with the rapid advance in next-generation sequencing in recent years. We supposed that the nasal NO screening plus genetic tests will be a feasible strategy for detecting PCD in patients with clinical manifestations suggesting this disease, considering the HSVA and TEM facilities are not available in vast majority of healthcare settings in China. We believe that accurate and prompt identification of PCD in patients who have clinical features compatible with this disease, will tailor treatment appropriately and allow counselling for this multisystem inherited disease.

Our systematic review has several strengths and limitations. We, for the first time, summarized the cases of PCD in Chinese populations based on available literatures, and compared the clinical characteristics of Chinese patients stratified by age at diagnosis, and further compared these features with Caucasian PCD patients, and highlighted the significant heterogeneity of clinical manifestations and genetic spectrum in Chinese patients, and demonstrated the adverse effect of a delayed diagnosis of PCD. However, all included studies were case reports and/or case series, in which we did not evaluate the study quality due to a lack of accepted assessment tool and could not exclude recall or ascertainment or selection bias. In addition, some findings of our study should be interpreted with caution because of a very small sample size and the lack of standardized data collection of included studies although our systematic review represents the most comprehensive descriptions of PCD in China to date. Another issue was the limited clinical data, such missing or unreliable information due to retrospective data collection and the lack of longitudinal information in our systematic review, that could be used to explore phenotype-genotype associations. It would therefore be essential for future studies to include longitudinal data to understand time-varying associations between phenotypes and genotypes in large collaborative clinical and research networks with standardized data collection in China in light of high variations of disease progression in PCD.

## Conclusion

The diagnosis of PCD in China was often delayed, which contributed to adverse health effects. Consistent with Caucasian patients, a significant heterogeneity of clinical and genetic characteristics was also found in Chinese patients. Our study highlights the importance of raising awareness among physicians and establishing the specialized referral centers to prompt early diagnosis and treatment of PCD. Fortunately, the national registration of rare diseases, including PCD, have been launched in China, which will undoubtedly bring the infinite hopes to Chinese PCD patients in the foreseeable future.

## Supplementary Information

**Additional file 1**. **Table S1.** Excluded articles after full-text review. **Table S2.** Study characteristics of included studies. **Table S3.** Clinical characteristics and laboratory results of patients who have a clinical diagnosis of PCD in excluded studies after full-text review.

## Data Availability

The data sets used and/or analyzed during the current study are available from the corresponding author on reasonable request.

## Abbreviations

PCD: Primary ciliary dyskinesia
TEM: Transmission electron microscopy
PA: Pseudomonas aeruginosa
NRDS: Neonatal respiratory distress syndrome
ERS: European Respiratory Society
ATS: American Thoracic Society
nNO: Nasal nitric oxide
HSVA: High-speed video analysis
SD: Standard deviation
IQR: Interquartile range
IDA: Inner dynein arm
ODA: Outer dynein arm
MTD: Microtubular disorganization
N-DRCs: Nexin link dynein regulator complex proteins
CA: Central apparatus
FEV_1_: Forced expiratory volume in one second
FVC: Forced vital capacity
SIT: Situs inversus totalis
HTX: Heterotaxy
SS: Situs solitus
Homo: Homozygous
Comp het: Compound heterozygous
ENT: Ear-nose-throat department
CHD: Congenital heart disease
OMIM: Online Mendelian Inheritance in Man
