# Supplementary material for: Clinical and genetic spectrum of primary ciliary dyskinesia in Chinese patients: a systematic review
Source: Orphanet J Rare Dis. 2022 Jul 19;17:283. doi: 10.1186/s13023-022-02427-1 (PMC9295413; doi:10.1186/s13023-022-02427-1)
Supplement: Supplementary file 1 — Additional file 1. Table S1. Excluded articles after full-text review. Table S2. Study characteristics of included studies. Table S3. Clinical characteristics and laboratory results of patients who have a clinical diagnosis of PCD in excluded studies after full-text review. [file 13023_2022_2427_MOESM1_ESM.docx]

| Author | Title | Journal | Year | Volume  (Issue) | Pages | Number of Patients |
| --- | --- | --- | --- | --- | --- | --- |
| Niu ZH et al | A sperm viability test using SYBR-14/propidium iodide flow cytometry as a tool for rapid screening of primary ciliary dyskinesia patients and for choosing sperm sources for intracytoplasmic sperm injection | Fertility and Sterility | 2011 | 95(1) | 389-392 | No detailed information |
| Shoemark A et al | High prevalence of CCDC103 p.His154Pro mutation causing primary ciliary dyskinesia disrupts protein oligomerisation and is associated with normal diagnostic investigations | Thorax | 2018 | 73(2) | 157-166 | Not in Chinese patients |
| Xia P et al | Whole-exome sequencing identify mutations in DNAH5 in a Chinese Han patient with primary ciliary dyskinesia: A case report | International Journal of Clinical and Experimental Medicine | 2018 | 11(8) | 8757-8761 | 1 |
| Guo Z et al | Clinical and genetic analysis of patients with primary ciliary dyskinesia caused by novel DNAAF3 mutations | Journal of Human Genetics | 2019 | 64(8) | 711-719 | 4 |
| Morimoto K et al | Recurring large deletion in DRC1 (CCDC164) identified as causing primary ciliary dyskinesia in two Asian patients | Mol Genet Genomic Med | 2019 | 7(8) | e838 | Not in Chinese patients |
| Zhang X et al | The value of nasal nitric oxide measurement in the diagnosis of primary ciliary dyskinesia | Pediatric Investigation | 2019 | 3(4) | 209-213 | 36 |
| Chen W et al | Comorbidities in situs inversus totalis: A hospital-based study | Birth Defects Research | 2020 | 112(5) | 418-426 | No detailed information |
| Sha Y et al | Biallelic mutations of CFAP74 may cause human primary ciliary dyskinesia and MMAF phenotype | Journal of Human Genetics | 2020 | 65(11) | 961-969 | 2 |
| Tu C et al | Novel mutations in SPEF2 causing different defects between flagella and cilia bridge: the phenotypic link between MMAF and PCD | Human Genetics | 2020 | 139(2) | 257-271 | 6 |
| Wang B et al | Double lung transplantation for end-stage Kartagener syndrome: A case report and literature review | Journal of Thoracic Disease | 2020 | 12(4) | 1588-1594 | 1 |
| Guo T et al | Bi-allelic BRWD1 variants cause male infertility with asthenoteratozoospermia and likely primary ciliary dyskinesia | Human Genetics | 2021 | 140(5) | 761-773 | 3 |
| Huang SG et al | Kartagener’s syndrome and mucociliary clearance | Chinese Journal of Tuberculosis and Respiratory Diseases | 1993 | 16(5) | 302 | 1 |
| Yen TH et al | Kartagener syndrome—a case report | Journal of Otolaryngology Medical Association | 1997 | 32(5) | 450-454 | 1 |
| Ma YY et al | A study on the relationship between the recurrent respiratory infection and ciliary structure abnormality | Chinese Journal of Practical Pediatrics | 2001 | 16(7) | 405-407 | No detailed information |
| Mou JH et al | Diagnosis of immotile cilia syndrome: Analysis of three cases | Journal of Clinical Pediatrics | 2002 | 20(9) | 554-555,557 | 3 |
| Yue HX et al | Azoospermia in two brothers with Kartagener syndrome：a family history and diagnosis. | Journal of Reproductive Medicine | 2008 | 17(z1) | 82-85 | 2 |
| Xu BP et al | The axonemal dynein intermediate chain 1 gene (DNAI 1) and axonemal dynein heavy chain 5 gene (DNAH5) study in children with Kartagener syndrome | Chinese Journal of Practical Pediatrics | 2008 | 23(12) | 934-937+970-971 | 4 |
| Wang ZB et al | A case report of primary ciliary dyskinesia | Journal of Shanxi Medical University | 2010 | 41(2) | 183-184 | 1 |
| Tian ZR et al | Nursing care of a patient with primary ciliary dyskinesia and aspirin intolerance syndrome receiving nasal endoscopy | Journal of Nursing Science | 2011 | 26(12) | 44-46 | 1 |
| Feng WJ et al | Familial primary ciliary dyskinesia: report of 2 cases and literatures review | China Journal of Endoscopy | 2013 | 19(4) | 445-448 | 2 |
| Zhang J et al | Gene diagnosis in patients with Kartagener syndrome induced chronic secretory otitis media | Chinese Journal of Otology | 2014 | 12(1) | 41-44 | 1 |
| Wen XY et al | CCDC40 mutation as a cause of primary ciliary dyskinesia: a case report and review of literature | Journal of Reproductive Medicine | 2015 | 24(11) | 942-945 | 1 |
| Zhou CM et al | Transmission electron microscope observation of primary ciliary dyskinesia | Journal of Hebei Medical University | 2015 | 36(7) | 810-811 | No detailed information |
| Hu XY et al | Primary ciliary dyskinesia: a case report and literature review | Progress in Modern Biomedicine | 2017 | 17(19) | 3681-3684 | 1 |
| Luo SJ et al | Kartagener syndrome: a case report and literature review | Journal of Clinical Research | 2017 | 34(12) | 2496 | 1 |
| Bao YL et al | Clinical characteristics of bronchiectasis in children | Journal of Nanjing Medical University (Natural Science) | 2018 | 38(12) | 1784-1786 | No detailed information |
| Cheng KB et al | Clinical analysis on 41 cases of Kartagener syndrome | Shaanxi Medical Journal | 2019 | 48(7) | 856-859,899 | 41 |
| Tian XL et al | The clinical characteristics of 17 cases of primary ciliary dyskinesia | Chinese Journal of Tuberculosis and Respiratory Diseases | 2017 | 40(4) | 278-283 | 17 |
| O'Callaghan C et al | High prevalence of primary ciliary dyskinesia in a British Asian population | Arch Dis Child | 2010 | 95(1) | 51-52 | Not in Chinese patients |
| Guan WJ et al | Next-generation sequencing for identifying genetic mutations in adults with bronchiectasis | Journal of Thoracic Disease | 2018 | 10(5) | 2618-2630 | No detailed information |

**Table S1: Excluded articles in full-text review**

| First author [ref.] | Year of publication | Number of Patients | Population | Study design | diagnostics |
| --- | --- | --- | --- | --- | --- |
| Tseng-Kai Lin [16] | 1998 | 1 | adult | Case report | Kartagener + TEM |
| Tsang KW [17] | 1998 | 5 | Children + adult | Case series | Kartagener + TEM + HSVA |
| Bi J [18] | 2010 | 2 | adult | Case report | Clinical suspicion + TEM |
| Tang X [19] | 2013 | 1 | Children | Case report | Kartagener + TEM |
| Chen W [20] | 2014 | 1 | adult | Case report | Clinical suspicion + TEM |
| Zhang J [21] | 2014 | 1 | adult | Case report | Clinical suspicion + genetic analysis |
| Cao Y [22] | 2016 | 3 | Children + adult | Case series | Clinical suspicion + TEM |
| Sui W [23] | 2016 | 1 | adult | Case report | Clinical suspicion + TEM + genetic analysis |
| Guo T [24] | 2017 | 6 | adult | Case series | Clinical suspicion + genetic analysis |
| Hou J [25] | 2017 | 1 | adult | Case report | Clinical suspicion + TEM |
| Xu X [26] | 2017 | 2 | Children | Case report | Kartagener + TEM + genetic analysis |
| Liu L [27] | 2018 | 1 | adult | Case report | Clinical suspicion + genetic analysis |
| Yang L [28] | 2018 | 2 | adult | Case report | Clinical suspicion + TEM + genetic analysis |
| Li P [29] | 2019 | 1 | Children | Case report | Clinical suspicion + genetic analysis |
| Yue Y [30] | 2019 | 5 | adult | Case–control | Kartagener + TEM + genetic analysis |
| Zhang W [31] | 2019 | 1 | Children | Case report | Kartagener + genetic analysis |
| Deng S [32] | 2020 | 1 | adult | Case report | Kartagener + genetic analysis |
| Wang Y [33] | 2020 | 2 | adult | Case–control | Clinical suspicion + TEM + genetic analysis |
| Sun M [34] | 2020 | 1 | adult | Case report | Clinical suspicion + genetic analysis |
| Huang C [35] | 2021 | 2 | Children + adult | Case report | Clinical suspicion + TEM + genetic analysis |
| Zhou L [36] | 2020 | 5 | adult | Case series | Kartagener + TEM + genetic analysis |
| Guo Z [12] | 2020 | 50 | Children | Case series | Clinical suspicion + TEM + HSVA + genetic analysis |
| Guan Y [13] | 2021 | 75 | Children | Case series | Clinical suspicion + TEM + genetic analysis |
| Li Y [37] | 2021 | 1 | adult | Case report | Clinical suspicion + genetic analysis |
| Chen Y [38] | 1995 | 1 | adult | Case report | Clinical suspicion + TEM |
| Zhou B [39] | 2001 | 1 | Children | Case report | Kartagener + TEM |
| Peng DH [40] | 2005 | 1 | Children | Case report | Clinical suspicion + TEM |
| Qin T [41] | 2006 | 1 | Children | Case report | Clinical suspicion + TEM |
| Wei YX [42] | 2007 | 2 | Children + adult | Case report | Clinical suspicion + TEM |
| Xu BP [43] | 2008 | 26 | Children | Case series | Clinical suspicion + TEM |
| Jin BB [44] | 2010 | 4 | Children + adult | Case report | Clinical suspicion + TEM |
| Dong YQ [45] | 2011 | 1 | adult | Case report | Clinical suspicion + TEM |
| Wang T [46] | 2011 | 1 | adult | Case report | Clinical suspicion + TEM |
| Liu SH [47] | 2012 | 1 | Children | Case report | Kartagener + TEM |
| Qu MY [48] | 2013 | 2 | adult | Case report | Clinical suspicion + TEM |
| Bai Y [49] | 2014 | 2 | adult | Case series | Clinical suspicion + genetic analysis |
| Nie HP [50] | 2014 | 1 | adult | Case report | Clinical suspicion + TEM |
| Jin YT [51] | 2015 | 4 | Children | Case series | Kartagener + TEM |
| Ren JY [52] | 2015 | 1 | adult | Case report | Kartagener + TEM |
| Wang YK [53] | 2015 | 1 | Children | Case report | Kartagener + TEM |
| Qiu X [54] | 2016 | 1 | adult | Case report | Clinical suspicion + TEM |
| Qi YY [55] | 2016 | 1 | Children | Case report | Kartagener + TEM + genetic analysis |
| Chen LL [56] | 2017 | 1 | Children | Case report | Clinical suspicion + TEM + genetic analysis |
| Chen TD [57] | 2018 | 2 | Children | Case report | Clinical suspicion + TEM + genetic analysis |
| Wang K [58] | 2018 | 3 | Children | Case report | Clinical suspicion + TEM + genetic analysis |
| Shen N [59] | 2019 | 1 | Children | Case report | Clinical suspicion + genetic analysis |
| Wang WM [60] | 2019 | 1 | Children | Case report | Kartagener + genetic analysis |
| Yang JJ [61] | 2019 | 1 | adult | Case report | Clinical suspicion + TEM |
| Yang Q [62] | 2019 | 1 | Children | Case report | Clinical suspicion + TEM + genetic analysis |
| Wang S [63] | 2020 | 1 | adult | Case report | Clinical suspicion + TEM + genetic analysis |
| Li Y [64] | 2020 | 2 | Children | Case report | Clinical suspicion + TEM + genetic analysis |
| Bin ST [65] | 2020 | 8 | Children | Case series | Clinical suspicion + TEM + genetic analysis |

**Table S2. Study characteristics of included studies**

**Table S3. Clinical characteristics and laboratory results of patients who have a clinical diagnosis of PCD in excluded studies after full-text review.**

| Parameters | Total  (n=130) | Age at diagnosis＜18  (n=49) | Age at diagnosis≥18  (n=81) | P values |
| --- | --- | --- | --- | --- |
| Age at diagnosis(n=118), mean (SD) | 26.0±17.3 | 7.8±3.5(n=45) | 37.3±11.9(n=73) | <0.001 |
| Gender, Female (%) | 52(40) | 17(34.7) | 35(43.2) | 0.34 |
| Age at symptom onset |  |  |  |  |
| Infancy | 49(37.7) | 44(89.8) | 5(6.2) | <0.0001 |
| Childhood | 79(60.8) | 2(4.1) | 77(95.1) | <0.0001 |
| Adulthood | 2(1.5) | NA | 2(2.5) | NA |
| Situs inversus totalis/heterotaxy | 69(53.1) | 15(30.6) | 54(66.7) | <0.0001 |
| Family history |  |  |  |  |
| Consanguineous parents (68) | 12(9.2) | 1(2) | 11(13.6) | 0.06 |
| PCD family history (64) | 13(10) | 1(2) | 12(14.8) | 0.04 |
| Infertility history |  |  |  |  |
| Female (2) | 1(0.8) | NA | 1(1.2) | NA |
| Male (45) | 22(16.9) | NA | 22(27.2) | NA |
| Symptoms and Comorbidities |  |  |  |  |
| Neonatal respiratory distress (40) | 12(9.2) | 12(24.5) | 0 | <0.0001 |
| Chronic wet cough (130) | 124(95.4) | 48(98) | 76(93.8) | 0.51 |
| Sinusitis (130) | 125(96.2) | 47(95.9) | 78(96.3) | 0.91 |
| Otitis media (17) | 15(11.5) | 4(8.2) | 11(13.6) | 0.35 |
| Hearing impairment (26) | 7(5.4) | 1(2) | 6(7.4) | 0.36 |
| Asthma (43) | 1(0.8) | 0 | 1(1.2) | 1.0 |
| Congenital heart disease (43) | 4(3.1) | 4(8.2) | 0 | 0.04 |
| Nasal NO test (n=45) |  | 39 | 6 |  |
| Below diagnostic cut-off | 38(84.4) | 34(87.2) | 4(66.7) | 0.23 |
| Respiratory culture (n=58) |  |  | 58 |  |
| Haemophilus influenzae | 9 | NA | 9(15.5) | NA |
| Pseudomonas aeruginosa | 21 | NA | 21(36.2) | NA |
| Streptococcus pneumoniae | 1 | NA | 1(1.7) | NA |
| Streptococcus viridans | 1 | NA | 1(1.7) | NA |
| Staphylococcus aureus | 3 | NA | 3(5.2) | NA |
| Klebsiella pneumoniae | 6 | NA | 6(10.3) | NA |
| Legionella pneumophila | 1 | NA | 1(1.7) | NA |
| Acinetobacter baumannii | 3 | NA | 3(5.2) | NA |
| Monilia albican | 3 | NA | 3(5.2) | NA |
| Aspergillus | 3 | NA | 3(5.2) | NA |
| normal flora | 10 | NA | 10(17.2) | NA |
| Radiographic features (n=130) |  | 49 | 81 |  |
| Bronchiectasis | 116(89.2) | 37(75.5) | 79(97.5) | <0.0001 |
| Atelectasis | 1(0.8) | 1(2) | 0 | 0.38 |
| Pneumonia | 11(8.5) | 9(18.4) | 2(2.5) | 0.01 |
| Nearly normal | 2(1.5) | 2(4.1) | 0 | 0.14 |
| Lung function |  |  |  |  |
| FEV1% of predicted, mean (SD) | 67.1±21(n=14) | 97.6±1.1 (n=2) | 62±18(n=12) | 0.02 |
| FVC % of predicted, mean (SD) | 73±17(n=12) | NA | 73±17(n=12) | NA |
